# P059. Neuropsychological assessment in a case of medication-overuse headache associated with probable executive deficit

**DOI:** 10.1186/1129-2377-16-S1-A154

**Published:** 2015-09-28

**Authors:** Alessandra Sansalone, Amerigo Costa, Rosario Iannacchero

**Affiliations:** Center for Headache and Adaptive Disorders, Unit of Neurology, Department of Neuroscience and Sense Organs, Azienda Ospedaliera “Pugliese-Ciaccio”, Catanzaro, Italy

## Background

Recent studies attest to a relationship between medication-overuse headache (MOH) and cognitive impairment, especially regarding visual memory, verbal memory, processing speed, decision-making and attention, which constitute the executive function (EF) [1]. EF impairment in MOH patients may relate to frontal cortex hypo-metabolism [2]. Evidence about this relationship is conflicting [3]. We present the case of a MOH patient showing, at a clinical-psychological evaluation, cognitive issues that prompted a neuropsychological assessment.

## Materials and methods

A 52-year-old woman with a 15-year clinical history of chronic migraine (CM) came to our Center in September 2014. She presented with daily migraine and continuous use of non-steroidal anti-inflammatory drugs (NSAIDs). Brain magnetic resonance imaging (RMI) showed an occipital arachnoid cyst. Venous magnetic resonance angiography (vMRA) was negative. At the headache assessment, the neurologist diagnosed probable MOH (pMOH). The clinical-psychological evaluation showed significant levels of anxiety (Zung Self-Rating Anxiety Scale = 45) and self-referred cognitive issues, especially regarding memory, attention and focusing. After three months, MOH diagnosis was confirmed. Given the cognitive symptoms, even though a preliminary assessment showed normal score (Cognitive Failure Questionnaire = 38), we performed a deeper neuropsychological assessment. We first assessed general cognitive efficiency (Mini Mental State Examination; Raven Progressive Matrices; Brief Intelligence Test); then, we tested memory (Corsi Test; words span test; brief text test; Rey Word Recognition test), and constructional praxis (Clock Drawing Test; Rey-Osterrieth Complex Figure). We also assessed EF (semantic and phonemic verbal fluency tests; Stroop Test; Trail Making Test; Wisconsin Card Sorting Test). Finally, we performed Minnesota Multiphasic Personality Inventory (MMPI-2), that excluded a structured psychopathology, and Baratt Impulsiveness Scale (BIS-11), that showed mild personality impulsivity. The patient had signed an informed consent to testing and research.

## Results

The neuropsychological profile was normal regarding: verbal memory; constructional praxis; selective attention; strategic planning; it was borderline regarding: general cognitive efficiency; abstract logical reasoning; prose memory; semantic fluency; spatial planning and attention; we detected deficits regarding: visual short-term memory; verbal long-term memory; visual long-term memory; phonemic fluency.

## Conclusions

The detected EF impairment could relate with behavioral aspects relevant to MOH, such as, non-adherence and compulsive medication use. The patient could benefit from psychological interventions complementary to the medical headache therapy: psycho-education might improve MOH management; problem-solving training might enhance health management strategies seeking; relaxation training might improve health-related anxiety. Functional neuroimaging is required to investigate the brain areas activity.

Written informed consent to publish was obtained from the patient(s).
